# Supplementary material for: 3D vector field-guided toolpathing for 3D bioprinting
Source: Commun Eng. 2025 Aug 14;4:154. doi: 10.1038/s44172-025-00489-0 (PMC12354870; doi:10.1038/s44172-025-00489-0)
Supplement: Supplementary file 2 — Supplementary Information [file 44172_2025_489_MOESM2_ESM.pdf]

Supplementary Information for: 3D vector field-guided toolpathing for 3D bioprinting

Meghan Rochelle Griffin[1*], Spencer E. Bertram[2*], Noah P. Robison[1], Angela Panoskaltsis-Mortari[3], Ravi Janardan[4], Michael C. McAlpine[2]

[1]Department of Biomedical Engineering, University of Minnesota, Minneapolis, Minnesota
[2]Department of Mechanical Engineering, University of Minnesota, Minneapolis, Minnesota
[3]Department of Pediatrics, University of Minnesota, Minneapolis, Minnesota
[4]Department of Computer Science & Engineering, University of Minnesota, Minneapolis, Minnesota
[*]These authors contributed equally

Contents

1 Supplementary Figures **3**

2 Supplementary Methods **10**
2.1 Notation . . . 10
2.2 NAATIV3 . . . 10
2.3 Tractography . . . 11
2.4 Sweep exclusion . . . 13
2.4.1 Cylinder/streamline intersection . . . 15
2.4.2 Infinite line/finite line segment distance minimization . . . 17
2.4.3 Minimum average direct-flip (MDF) distance . . . 19
2.4.4 Initial path selection . . . 19
2.5 Cut plane . . . 20
2.6 Dependency graph population . . . 22
2.7 Maximum acyclic subgraph selection . . . 25
2.7.1 Alternative heuristic methods and performance comparison . . . 27
2.8 Ordering . . . 28
2.9 Cardiac Toolpathing . . . 30
2.9.1 Algorithm performance . . . 31

List of Algorithms

1 NAATIV3 . . . 11
2 4th order Runge-Kutta tractography . . . 12
3 4th order Runge-Kutta propagation . . . 13
4 Sweep exclusion . . . 15
5 Streamline/cylinder intersection procedure . . . 16
6 Infinite line/finite segment distance minimization . . . 18

7 MDF-based next path selection 19
8 Splitting streamlines via a cut plane 21
9 Dependency graph population 23
10 Interference prediction 25
11 Maximum acyclic subgraph selection 25
12 Directed graph bidirectional edge removal 26
13 Iterative stochastic maximum acyclic subgraph selection 27
14 Greedy search toolpath ordering algorithm 30

1 Supplementary Figures

Supplementary Table 1. Medical history for the two ex vivo hearts used in the development of this algorithm.

Heart	Sex	Age	BMI	Cardiac History	Other Medical History	Cause of Death
"Healthy"	Male	60	26.3	None known	None known	Stroke
"Diseased"	Male	50	31.1	Coronary artery disease, hypercholesterolemia, hypertension	Smoking	Myocardial infarction

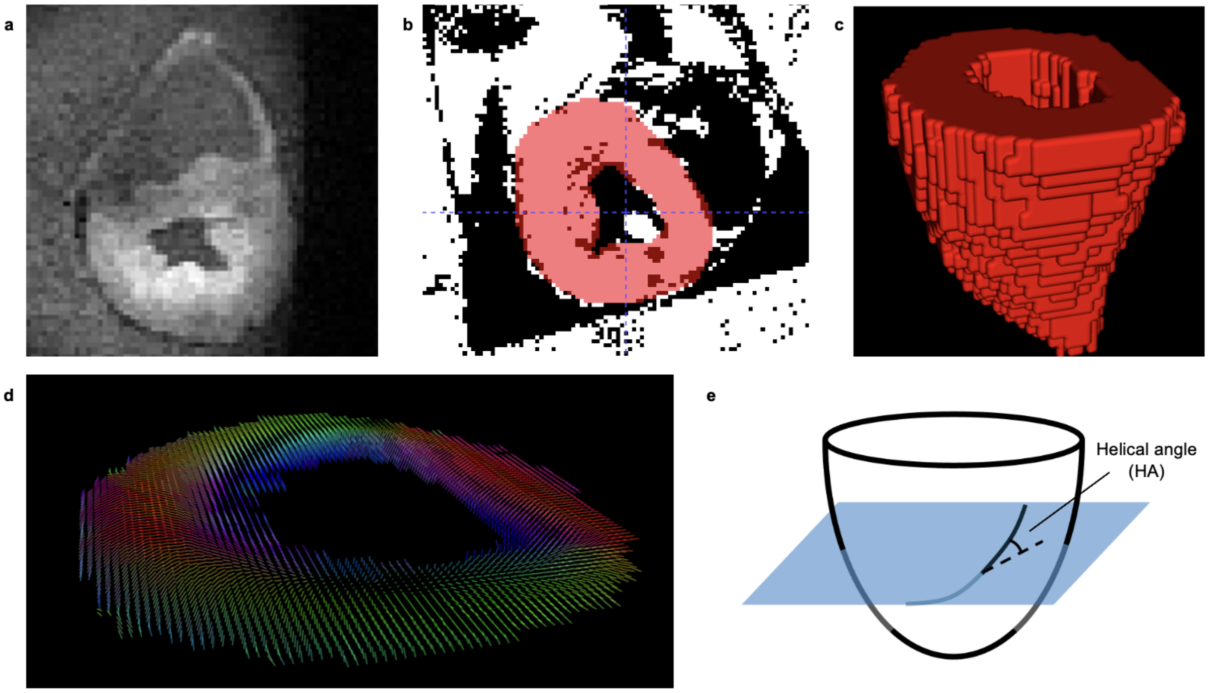

Supplementary Figure 1. a) Cross section of raw DTMRI data from a human heart. b) Example of creating the mask for the left ventricle in ITK Snap. Mask is shown in red. c) 3D rendering of the voxel mask created to isolate the left ventricle, shown with a slight smoothing. d) Short-axis cross section of the fiber orientation field, obtained from the major eigenvector of the diffusion tensor. e) Definition of helical angle, the angle between a myofiber and the short-axis plane.

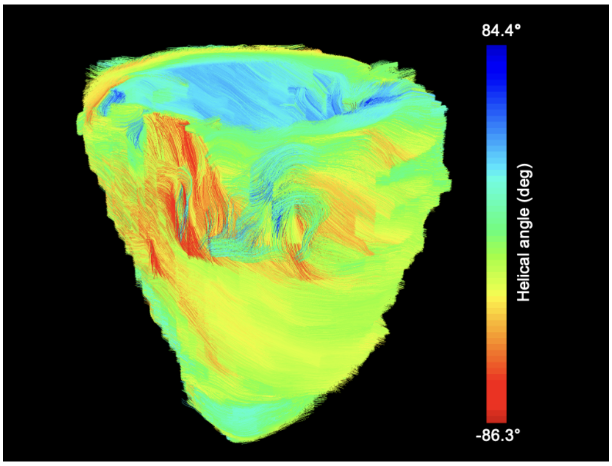

Supplementary Figure 2. Complete tractography results for the isolated left ventricle of a human heart post myocardial infarction.

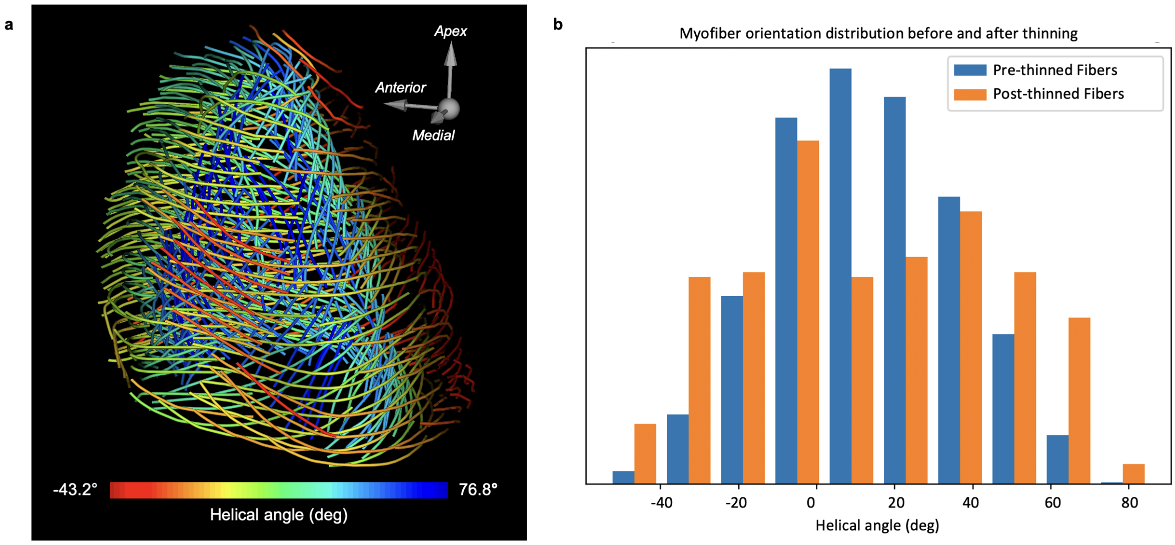

Supplementary Figure 3. a) Thinned streamline set from 1:4 scale healthy human left ventricle model, color coded by helical angle. b) Normalized helical angle histogram before and after thinning of 1:4 scale healthy human left ventricle model.

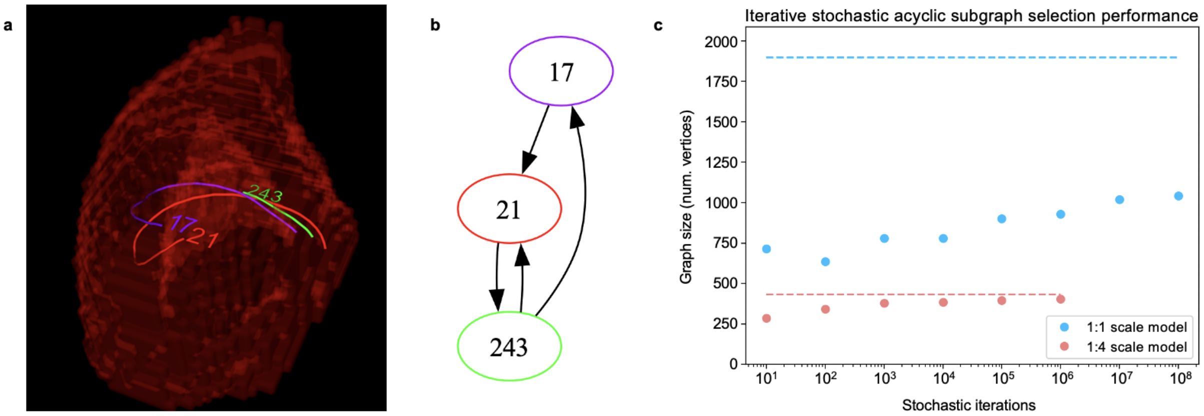

Supplementary Figure 4. a) 3D rendering of a dependency graph cycle from the 1:4 scale toolpath data. Bounding volume shown in red. b) Directed dependency graph associated with (a). c) Dependency graph toolpath (vertex) retainment during acyclic subgraph selection as a function of stochastic iterations. Initial graph size denoted by dashed lines.

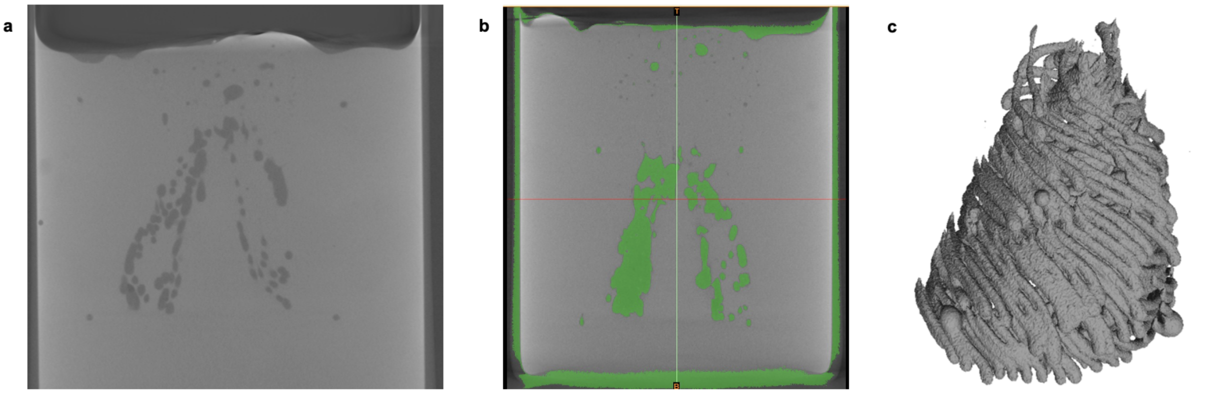

Supplementary Figure 5. a) Representative slice of raw micro-CT data. b) Thresholding segmentation shown on a representative slice of micro-CT data. Example shown on a different slice than shown in (a). c) Isometric view of 3D model generated from micro-CT segmentation.

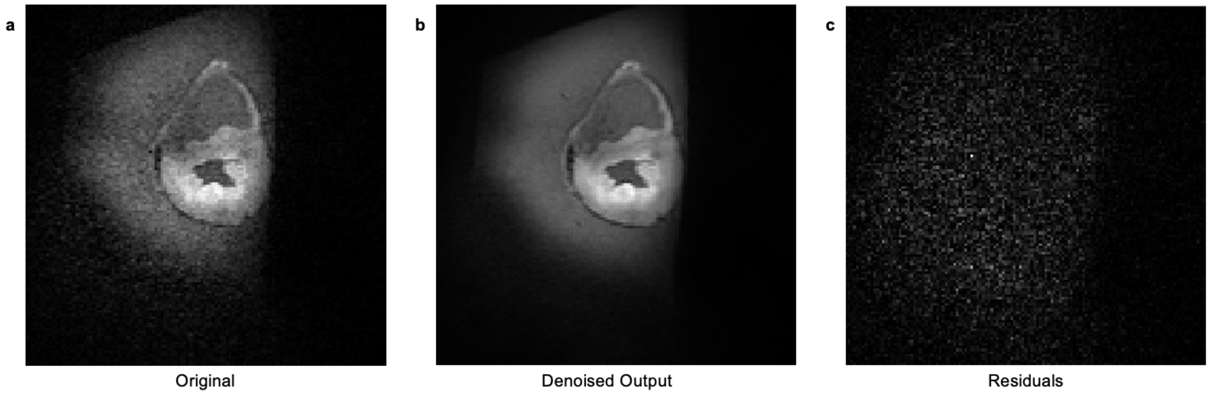

Supplementary Figure 6. Denoising of DTMRI data. Representative slice shown before (left) and after (center) denoising process. Residual plot (right) shows random noise, indicating no loss of structural data.

2 Supplementary Methods

2.1 Notation

A 3D contour representing a sequence of 3D points is often referred to as a streamline. For example, a streamline P consists of the ordered sequence of N 3D points $\{\boldsymbol{p}_i\}_1^N$. Other streamline labels (P_1, Q, etc.) are used to denote a streamline in various algorithm outlines and mathematical operations, which should be apparent from context. The $\frown$ operator is used to denote sequence concatenation, such that $P_1 \frown P_2$ denotes appending the point sequence P_2 to the end of P_1. This notation is also used to describe appending a single point to a point sequence via $P \frown \{\boldsymbol{p}\}$, which denotes appending the point $\boldsymbol{p}$ to the end of the point sequence P. As such, the number of points in P, denoted $|P|$, is incremented by 1 during this operation. The notation $P[i]$ is used to denote the ith term of the sequence, beginning with $P[1]$ and ending with $P[|P|]$. The last point in a sequence P may also be denoted by $P[-1]$.

An unordered set of streamlines is denoted S. For example, a set of N streamlines is described by $S = \{P_i\}$, where $|S| = N$. An ordered sequence of streamlines is denoted T. For example, a sequence of N streamlines is described by $T = \{P_i\}_1^N$.

The construction of a directed graph is denoted (V, E). For example, $D \leftarrow (V, E)$ denotes a directed graph with vertices V and edges (ordered pairs of vertices) E. The addition of an edge $e = \langle v_i, v_j \rangle$ to a graph is denoted $E(D) \cup \{e\}$, where $E(D)$ denotes the edge set of D. Additionally, the directed graphs described have vertex sets that one-to-one correspond to streamlines in a given set. In other words, a graph may be constructed via $D \leftarrow (S, \emptyset)$, denoting that each vertex in D corresponds to a streamline P in the unordered streamline set S. As such, vertices of D may be simply referred to by their corresponding streamline $P \in S$. For example, the edge $\langle P_i, P_j \rangle$ denotes a directed edge between the vertex corresponding to P_i and the vertex corresponding to P_j. Finally, the streamline set is synonymous to the vertex set of a directed graph D, such that $S \leftarrow V(D)$ represents extracting the streamlines corresponding to each vertex in $V(D)$, which are stored in the unordered streamline set S.

At various times, the term N is used to describe either the length of a streamline (the number of points comprising the point sequence), the size of an ordered sequence of streamlines, or the size of an unordered set of streamlines. At the highest level, N is an input parameter to NAATIV3, denoting the number of iterations to use for iterative stochastic maximum acyclic subgraph selection (section 2.7). The meaning of N in specific situations should be apparent from context.

2.2 NAATIV3

NAATIV3 takes as input a continuous $\mathbb{R}^3 \rightarrow \mathbb{R}^3$ vector field $\boldsymbol{v}(\boldsymbol{x})$ defined within some region of space M. The sole purpose of M is to define a binary mapping function in $\mathbb{R}^3$ that returns 1 if a point $\boldsymbol{x} \in M$, otherwise 0 if $x \notin M$ (see section 2.3). In this way, M effectively defines a binary partition of space, which in turn defines the geometry of the object to be 3D printed. M may be a discrete triangulated surface, an analytically defined region of space, or, in the context of biomedical imaging, a voxelized region of space (also referred to as a binary "mask").

The output of NAATIV3 is a sequence of directed 3D contours (point sequences) $T = \{P_i\}_{i=1}^N$, where each P_i consists of a sequence of 3D points $\{\boldsymbol{p}_{i,j}\}_{j=1}^{N_i}$ and N_i denotes the number of points in P_i. The sequence T defines a toolpath sequence, which can be converted into a required file format for 3D printing, such as G-code. A comprehensive overview of NAATIV3 is described in Algorithm 1. Note that the "cut plane" step is optional, depending on user judgment of the specific geometry of the input vector field and bounding volume.

Algorithm 1: NAATIV3

Input : M, bounding volume
$\boldsymbol{v}(\boldsymbol{x})$, continuous vector field defined within M
n_{seed}, seed density for tractography
l_{max}, maximum streamline length for tractography
Δs, step size for tractography
w_s, desired toolpath spacing
`cut_plane`, (optional) cut plane geometry
d_p, print line diameter
d_n, nozzle outer diameter
N, iterations for stochastic maximum acylic subgraph selection

Output: T, the ordered toolpath sequence

Function `NAATIV3`(M, $\boldsymbol{v}(\boldsymbol{x})$, n_{seed}, l_{max}, Δs, w_s, `cut_plane`, d_p, d_n, N):
  $S \leftarrow$ `tractography`(M, $\boldsymbol{v}(\boldsymbol{x})$, n_{seed}, l_{max}, Δs)
  $S_{thinned} \leftarrow$ `sweepExclusion`(S, w_s)
  initialize D `// dependency graph, storing the streamline associated with each vertex`
  if `cut_plane` *is provided* **then**
    S_{cut}, $C \leftarrow$ `planeCut`($S_{thinned}$, `cut_plane`)
    $D \leftarrow$ `populateDependencyGraph`(S_{cut}, C, d_p, d_n)
  else
    $D \leftarrow$ `populateDependencyGraph`($S_{thinned}$, `NULL`, d_p, d_n)
  end
  $D_{acyclic} \leftarrow$ `maximumAcyclicSubgraph`(D, N)
  $T \leftarrow$ `greedySearch`($D_{acyclic}$)
  return T

2.3 Tractography

NAATIV3 uses 4th order, fixed step size Runge-Kutta tractography [1] to generate the initial set of streamlines (Algorithm 2), implemented via a custom fork of the Diffusion Imaging in Python (DIPY) library [4]. To begin, a set of seed points with uniform spatial density n_{seed} is created throughout the bounding volume M. The specific implementation of seed point generation is dependent on the nature of M. In this work, M takes the form of a voxelized volume, throughout which seed points are generated in a uniform cubic lattice. In this context, n_{seed} defines the point density of the lattice in terms of $\frac{seeds}{voxel}$ or $\frac{seeds}{mm^3}$.

From each seed point $\boldsymbol{s}$, the initial direction vector $\boldsymbol{v}(\boldsymbol{s})$ is obtained and iteratively propagated through space according to Algorithm 3. Importantly, the sign of $\boldsymbol{v}(\boldsymbol{x})$ at any point is arbitrary; as such, two distinct streamlines are generated from each seed point, using both the "forward" and "backward" initial direction vectors $\boldsymbol{v}(\boldsymbol{s})$ and $-\boldsymbol{v}(\boldsymbol{s})$. For a set of N seed points, $2N$ streamlines are produced. The sign of $\boldsymbol{v}(\boldsymbol{x})$ is determined such that sense is retained during propagation. Propagation for a streamline is terminated upon reaching the specified maximum streamline length l_{max} or the boundary of the region M. Any streamline that falls under the minimum length l_{min} is removed from the set. Notably, it is assumed that $\boldsymbol{v}(\boldsymbol{x})$ represents a unit vector field, such that $||\boldsymbol{v}(\boldsymbol{x})|| = 1$ for all $\boldsymbol{x} \in M$. Such normalization is trivial in practice.

Algorithm 2: 4th order Runge-Kutta tractography

Input : M, bounding volume
$\boldsymbol{v}(\boldsymbol{x})$, continuous vector field defined within M
n_{seed}, seed density
l_{max}, maximum streamline length
Δs, propagation step size
Output: S, the produced set of streamlines

Function `tractography`(M, $\boldsymbol{v}(\boldsymbol{x})$, n_{seed}, l_{max}, Δs):
  initialize S
  for each $\boldsymbol{s}_i \in$ `generateSeedPoints`(M, n_{seed}) **do**
    for `sign` *in* $(-1, 1)$ **do** `// positive and negative initial vector`
      $P \leftarrow \{\boldsymbol{s}_i\}$ `// current streamline point sequence`
      while $P[|P|] \in M$ *AND* `euclideanLength`(P) $< l_{max}$ **do**
        if $|P| = 1$ **then** `// first point`
          $\boldsymbol{v}_{prop} \leftarrow$ `sign` $\cdot\ \boldsymbol{v}(\boldsymbol{s})$
          $\boldsymbol{p}_{next} \leftarrow \boldsymbol{s}_i + \boldsymbol{v}_{prop}\Delta s$
          $P \leftarrow P^\frown\{\boldsymbol{p}_{next}\}$ `// append point`
        else
          $\boldsymbol{v}_{prev} \leftarrow \frac{P[|P|]-P[|P|-1]}{||P[|P|]-P[|P|-1]||}$ `// normalized previous propagation vector`
          $\boldsymbol{v}_{prop} \leftarrow$ `propagate`($\boldsymbol{v}(\boldsymbol{x})$, $\boldsymbol{v}_{prev}$, $P[|P|]$, Δs)
          $\boldsymbol{p}_{next} \leftarrow P[|P|] + \boldsymbol{v}_{prop}\Delta s$
          $P \leftarrow P^\frown\{\boldsymbol{p}_{next}\}$ `// append point`
        end
      end
      $S \leftarrow S \cup \{P\}$
    end
  end
  return S

Algorithm 3: 4th order Runge-Kutta propagation

Input : $\boldsymbol{v}(\boldsymbol{x})$, continuous vector field
$\boldsymbol{v}_{prev}$, previous propagation vector (to retain sense)
$\boldsymbol{p}$, point from which to propagate
Δs, propagation step size

Output: $\boldsymbol{v}_p$, the unit propagation vector at $\boldsymbol{p}$

Function `propagate`($\boldsymbol{v}(\boldsymbol{x})$, $\boldsymbol{v}_{prev}$, $\boldsymbol{p}$, Δs):
- $\boldsymbol{k}_1 \leftarrow \boldsymbol{v}(\boldsymbol{p})$
- $\boldsymbol{k}_1 \leftarrow$ `sign`$(\boldsymbol{v}_{prev} \cdot \boldsymbol{k}_1)$ $\boldsymbol{k}_1$ `// retain sense`
- $\boldsymbol{k}_2 \leftarrow \boldsymbol{v}(\boldsymbol{p} + \frac{\Delta s}{2}\boldsymbol{k}_1)$
- $\boldsymbol{k}_2 \leftarrow$ `sign`$(\boldsymbol{k}_1 \cdot \boldsymbol{k}_2)$ $\boldsymbol{k}_2$ `// retain sense`
- $\boldsymbol{k}_3 \leftarrow \boldsymbol{v}(\boldsymbol{p} + \frac{\Delta s}{2}\boldsymbol{k}_2)$
- $\boldsymbol{k}_3 \leftarrow$ `sign`$(\boldsymbol{k}_2 \cdot \boldsymbol{k}_3)$ $\boldsymbol{k}_3$ `// retain sense`
- $\boldsymbol{k}_4 \leftarrow \boldsymbol{v}(\boldsymbol{p} + \Delta s \boldsymbol{k}_3)$
- $\boldsymbol{k}_4 \leftarrow$ `sign`$(\boldsymbol{k}_3 \cdot \boldsymbol{k}_4)$ $\boldsymbol{k}_4$ `// retain sense`
- $\boldsymbol{v}_p \leftarrow \frac{\boldsymbol{k}_1 + 2\boldsymbol{k}_2 + 2\boldsymbol{k}_3 + \boldsymbol{k}_4}{||\boldsymbol{k}_1 + 2\boldsymbol{k}_2 + 2\boldsymbol{k}_3 + \boldsymbol{k}_4||}$
- **return** $\boldsymbol{v}_p$

2.4 Sweep exclusion

The set of dense streamlines is reduced down to a representative subset, to be used directly as toolpaths, using a novel method termed "sweep exclusion" (Algorithm 4). The goal is to reduce the set down to a subset possessing uniform spatial covering, controlled inter-streamline spacing, and high average toolpath length. Sweep exclusion performs optimally given a highly dense and exactly parallel input streamline set (Suppl. Fig. 7). Therefore, sweep exclusion relies on the assumptions of a sufficiently dense and locally parallel initial streamline set, which is determined by the geometry of the NAATIV3 input vector field and bounding volume.

Sweep exclusion begins by selecting the longest path in the input streamline set S (see section 2.4.4 for further details regarding this feature). This streamline is set as the initial swept path, along which a swept cylinder is created with radius w_s. All other streamlines that intersect this cylinder are removed. The next path to sweep is then selected via minimum average direct-flip distance (see section 2.4.3), its swept cylinder is created, and all intersecting streamlines are removed. This process is repeated until all remaining streamlines have been swept.

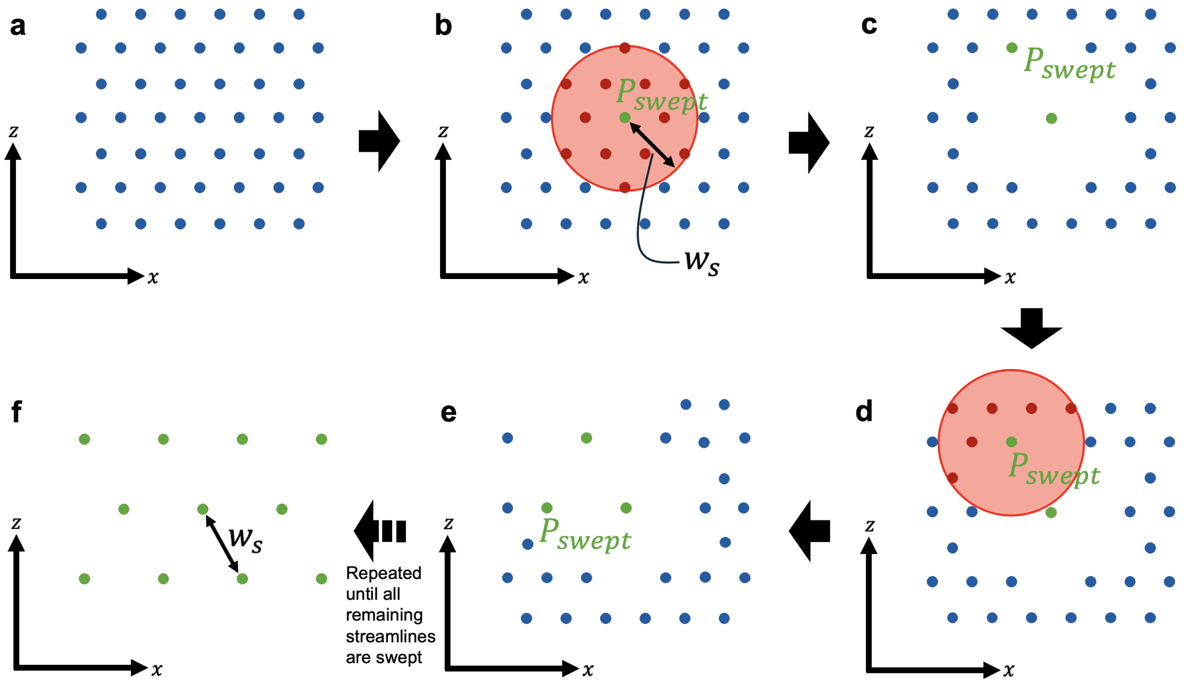

Supplementary Figure 7. 2D cross section (perpendicular to input 3D streamlines) of sweep exclusion operation under optimal input case, where the input streamline set is exactly parallel and uniformly dense. Unvisited paths shown in blue. Swept paths shown in green. Cylinder cross section and removed paths shown in red. a) 2D cross section of exactly parallel, uniformly dense input streamline set. b) initially selected swept path with swept cylinder of radius w_s. c) streamline set after first iteration, with the next swept path selected. d) second swept path selection and removal. e) streamline set after the second iteration, with the third swept streamline selected. f) the final streamline set after sweep exclusion, with uniform spacing w_s.

Algorithm 4: Sweep exclusion

Input : $S = \{P_i\}_1^N$, streamline set
w_s, ideal inter-streamline spacing
Output: $S_{thinned}$ the reduced representative subset of S

Function `sweepExclusion`(S, w_s):
  $S_{swept} \leftarrow \emptyset$
  initialize P_{swept}
  while $S - S_{swept} \neq \emptyset$ **do**
    if $S_{swept} = \emptyset$ **then** `// start with longest streamline`
      $P_{swept} \leftarrow$ `LongestPath`(S)
    else
      $P_{swept} \leftarrow$ `minMDF`(P_{swept}, $S - S_{swept}$)
    end
    $S_{swept} \leftarrow S_{swept} \cup \{P_{swept}\}$
    for each $P_i \in S - S_{swept}$ **do**
      if `streamCylInt`(P_i, Q, w_s) **then**
        $S \leftarrow S - \{P_i\}$
      end
    end
  end
  $S_{thinned} \leftarrow S$
  return $S_{thinned}$

2.4.1 Cylinder/streamline intersection

An efficient cylinder/streamline intersection procedure (Algorithm 5) determines whether a given streamline Q intersects the swept cylinder along another streamline P. Notably, this procedure is analytically based and does not involve creation of a cylinder mesh. The procedure operates segment-wise, comparing every pair of segments $\overline{\boldsymbol{p}_i\boldsymbol{p}_{i+1}}$ and $\overline{\boldsymbol{q}_j\boldsymbol{q}_{j+1}}$ in P and Q (Suppl. Fig. 8). Two "endplanes" are determined for each segment $\overline{\boldsymbol{p}_i\boldsymbol{p}_{i+1}}$, which serve to internally define the region of space spanned by the corresponding section of the swept cylinder. These endplanes are constructed by bisecting the current segment $\overline{\boldsymbol{p}_i\boldsymbol{p}_{i+1}}$ with the previous segment $\overline{\boldsymbol{p}_{i-1}\boldsymbol{p}_i}$ and the next segment $\overline{\boldsymbol{p}_{i+1}\boldsymbol{p}_{i+2}}$, such that the entire set of endplanes along a streamline P precisely partition the region of space spanned by the swept cylinder. Each segment $\overline{\boldsymbol{q}_j\boldsymbol{q}_{j+1}}$ is then effectively truncated to be within these endplanes by modifying the domain of the parametric function for the segment, $\boldsymbol{q}_j(t)$. A parametric function $\boldsymbol{p}_i(t)$ is similarly defined for the segment $\overline{\boldsymbol{p}_i\boldsymbol{p}_{i+1}}$. The minimum distance d_{min} between $\boldsymbol{q}_j(t)$ on the truncated domain $t \in [\tau_0, \tau_1]$ and $\boldsymbol{p}_i(s)$ on the infinite domain $s \in (-\infty, \infty)$ is found (see section 2.4.2). The infinite domain for s is necessitated by the skew nature of the cylinder segment (Suppl. Fig. 10). If d_{min} is less than the cylinder radius r, then $\overline{\boldsymbol{q}_j\boldsymbol{q}_{j+1}}$ must intersect the portion of the swept cylinder internal to the bisecting endplanes around $\overline{\boldsymbol{p}_i\boldsymbol{p}_{i+1}}$. Therefore Q must intersect the swept cylinder around P.

Algorithm 5: Streamline/cylinder intersection procedure

Input : $P = \{\boldsymbol{p}_i\}_1^N$, the cylinder central axis
$Q = \{\boldsymbol{q}_j\}_1^M$, the streamline to check for intersection
r, the cylinder radius
Output: True if Q intersects the swept cylinder along P, otherwise False

```
Function streamCylInt(P, Q, r):
    for i = 1 to N − 1 do
        // calculate first endplane normal n_i
        if i = 0 then // first segment
            n_i ← (p_i − p_{i+1}) / ||p_i − p_{i+1}||
        else
            v_{i,i+1} ← (p_{i+1} − p_i) / ||p_{i+1} − p_i||
            v_{i,i−1} ← (p_{i−1} − p_i) / ||p_{i−1} − p_i||
            b_i ← (v_{i,i+1} + v_{i,i−1}) / ||v_{i,i+1} + v_{i,i−1}|| // bisecting vector for first endplane
            n_i ← b_i × (v_{i,i+1} × v_{i,i−1})
        end
        // calculate second endplane normal n_{i+1}
        if i = N − 1 then // last segment
            n_{i+1} ← (p_{i+1} − p_i) / ||p_{i+1} − p_i||
        else
            v_{i+1,i} ← −v_{i,i+1}
            v_{i+1,i+2} ← (p_{i+1} − p_{i+1}) / ||p_{i+2} − p_{i+1}||
            b_{i+1} ← (v_{i+1,i} + v_{i+1,i+2}) / ||v_{i+1,i} + v_{i+1,i+2}|| // bisecting vector for second endplane
            n_{i+1} ← b_{i+1} × (v_{i+1,i+2} × v_{i+1,i})
        end
        p_i(s) := p_i + (p_{i+1} − p_i)s
        for j = 1 to M − 1 do
            q_j(t) := q_j + (q_{j+1} − q_j)t
            // find the intersections of q_j(t) with each endplane
            τ_0 ← t such that n_i · (p_i − q_j(t)) = 0
            τ_1 ← t such that n_{i+1} · (p_{i+1} − q_j(t)) = 0
            τ_0, τ_1 ← min(τ_0, τ_1), max(τ_0, τ_1)
            if τ_0 ∈ [0, 1] OR τ_1 ∈ [0, 1] then // part of segment lies between endplanes
                τ_0 ← max(τ_0, 0)
                τ_1 ← min(τ_1, 1)
                d_min ← minDistance(p_i, p_{i+1}, q_j(τ_0), q_j(τ_1))
                if d_min ≤ r then
                    return True
                end
            end
        end
    end
    return False
```

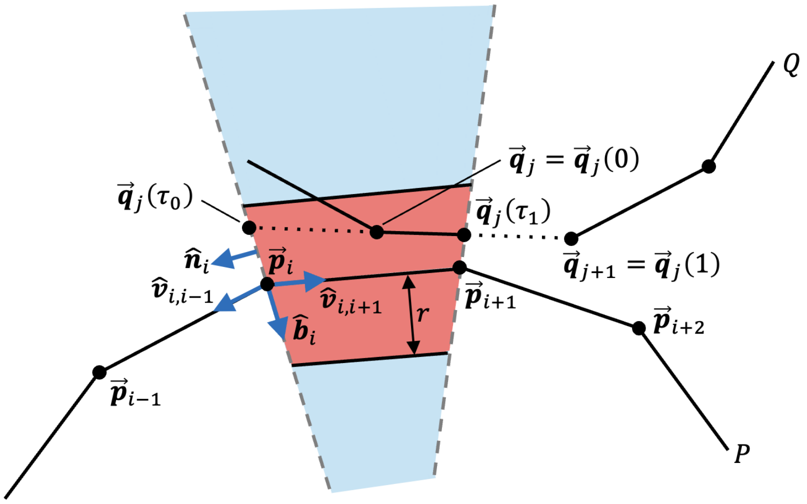

Supplementary Figure 8. 2D schematic of 3D cylinder/streamline intersection procedure. Region between skew cylinder segment endplanes shown in light blue. Region encompassed by skew cylinder shown in red. Bisecting and normal vectors defining the endplanes denoted by $\hat{\boldsymbol{b}}$ and $\hat{\boldsymbol{n}}$, respectively.

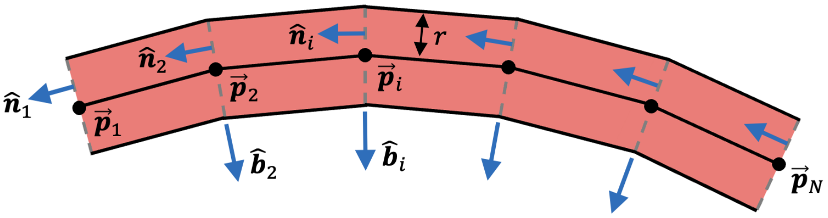

Supplementary Figure 9. 2D schematic of swept cylinder of radius r created around a streamline P, defined segmentwise. Bisecting planes shown in dashed grey, with normal vectors $\boldsymbol{n}_i$ and bisecting vectors $\boldsymbol{b}_i$ shown in blue. Note that in Algorithm 5, the normals $\boldsymbol{n}_i$ and $\boldsymbol{n}_{i+1}$ for a given segment point outward from the segment. Also, no bisecting vector is defined for $\boldsymbol{p}_1$ or $\boldsymbol{p}_N$; instead, $\boldsymbol{n}_0$ and $\boldsymbol{n}_N$ are taken to be parallel to the first and last segment, respectively.

2.4.2 Infinite line/finite line segment distance minimization

Here, a generalized description of the infinite line/finite line segment euclidean distance minimization function used in Algorithm 5 is provided. The procedure is described in full in Algorithm 6. Given an infinite line containing two points $\boldsymbol{a}_0$ and $\boldsymbol{a}_1$, and a line segment defined by two endpoints $\boldsymbol{b}_0$ and $\boldsymbol{b}_1$, the minimum distance between them can be found via minimization of the squared Euclidean distance function $D(s,t) = ||\boldsymbol{a}(s) - \boldsymbol{b}(t)||^2$ on the domain $s \in (-\infty, \infty)$, $t \in [0,1]$, where $\boldsymbol{a}(s) = \boldsymbol{a}_0 + (\boldsymbol{a}_1 - \boldsymbol{a}_0)s$ and $\boldsymbol{b}(t) = \boldsymbol{b}_0 + (\boldsymbol{b}_1 - \boldsymbol{b}_0)t$. $D(s,t)$ can be expanded to

$$D(s,t) = C_1 s^2 - 2C_2 st + C_3 t^2 + 2C_4 s - 2C_5 t + C_6 \quad (1)$$

where $C_1 = (\boldsymbol{a}_1 - \boldsymbol{a}_0)\cdot(\boldsymbol{a}_1 - \boldsymbol{a}_0)$, $C_2 = (\boldsymbol{a}_1 - \boldsymbol{a}_0)\cdot(\boldsymbol{b}_1 - \boldsymbol{b}_0)$, $C_3 = (\boldsymbol{b}_1 - \boldsymbol{b}_0)\cdot(\boldsymbol{b}_1 - \boldsymbol{b}_0)$, $C_4 = (\boldsymbol{a}_1 - \boldsymbol{a}_0)\cdot(\boldsymbol{a}_0 - \boldsymbol{b}_0)$, $C_5 = (\boldsymbol{b}_1 - \boldsymbol{b}_0)\cdot(\boldsymbol{a}_0 - \boldsymbol{b}_0)$, and $C_6 = (\boldsymbol{a}_0 - \boldsymbol{b}_0)\cdot(\boldsymbol{a}_0 - \boldsymbol{b}_0)$. The gradient is then given by:

$$\nabla D(s,t) = \langle \frac{\partial D}{\partial s}, \frac{\partial D}{\partial t} \rangle = 2\langle C_1 s - C_2 t + C_4, C_2 s + C_3 t - C_5 \rangle \quad (2)$$

which evaluates to $\mathbf{0}$ at the point $(\bar{s}, \bar{t}) = (\frac{C_2C_5 - C_3C_4}{C_1C_3 - C_2^2}, \frac{C_1C_5 - C_2C_4}{C_1C_3 - C_2^2})$. If $\bar{t} \in [0, 1]$, the minimum distance is found simply via $d_{min} = \sqrt{D(\bar{s}, \bar{t})}$. If $\bar{t} > 1$, then the minimimum of D on the domain $s \in (-\infty, \infty)$, $t \in [0, 1]$ must lie at the point $(s_1, 1)$ such that $\frac{\partial D(s_1,1)}{\partial s} = 0$, yielding $s_1 = \frac{C_2 - C_4}{C_1}$ and $d_{min} = \sqrt{D(s_1, 1)}$. Otherwise, if $\bar{t} < 0$, then the minimum of D on the restricted domain must lie at the point $(s_0, 0)$ such that $\frac{\partial D(s_0,0)}{\partial s} = 0$, yielding $s_0 = \frac{-C_4}{C_1}$ and $d_{min} = \sqrt{D(s_0, 0)}$.

Algorithm 6: Infinite line/finite segment distance minimization

Input : $\boldsymbol{a}_0$, a point on the infinite line,
$\boldsymbol{a}_1$, another point on the infinite line,
$\boldsymbol{b}_0$, the first endpoint of the line segment,
$\boldsymbol{b}_1$, the second endpoint of the line segment

Output: d_{min}, the minimum euclidean distance between the infinite line $\overleftrightarrow{\boldsymbol{a}_0\boldsymbol{a}_1}$ and the line segment $\overline{\boldsymbol{b}_0\boldsymbol{b}_1}$

Function `minDistance`($\boldsymbol{a}_0$, $\boldsymbol{a}_1$, $\boldsymbol{b}_0$, $\boldsymbol{b}_1$):
- $C_1 \leftarrow (\boldsymbol{a}_1 - \boldsymbol{a}_0) \cdot (\boldsymbol{a}_1 - \boldsymbol{a}_0)$
- $C_2 \leftarrow (\boldsymbol{a}_1 - \boldsymbol{a}_0) \cdot (\boldsymbol{b}_1 - \boldsymbol{b}_0)$
- $C_3 \leftarrow (\boldsymbol{b}_1 - \boldsymbol{b}_0) \cdot (\boldsymbol{b}_1 - \boldsymbol{b}_0)$
- $C_4 \leftarrow (\boldsymbol{a}_1 - \boldsymbol{a}_0) \cdot (\boldsymbol{a}_0 - \boldsymbol{b}_0)$
- $C_5 \leftarrow (\boldsymbol{b}_1 - \boldsymbol{b}_0) \cdot (\boldsymbol{a}_0 - \boldsymbol{b}_0)$
- $C_6 \leftarrow (\boldsymbol{a}_0 - \boldsymbol{b}_0) \cdot (\boldsymbol{a}_0 - \boldsymbol{b}_0)$
- $\bar{s} \leftarrow \frac{C_2C_5 - C_3C_4}{C_1C_3 - C_2^2}$
- $\bar{t} \leftarrow \frac{C_1C_5 - C_2C_4}{C_1C_3 - C_2^2}$
- **if** $\bar{t} < 0$ **then**
  - $s_0 \leftarrow \frac{-C_4}{C_1}$
  - **return** $\sqrt{D(s_0, 0)}$
- **else if** $0 \leq \bar{t} \leq 1$ **then**
  - **return** $\sqrt{D(\bar{s}, \bar{t})}$
- **else**
  - $s_1 \leftarrow \frac{C_2 - C_4}{C_1}$
  - **return** $\sqrt{D(s_1, 1)}$
- **end**

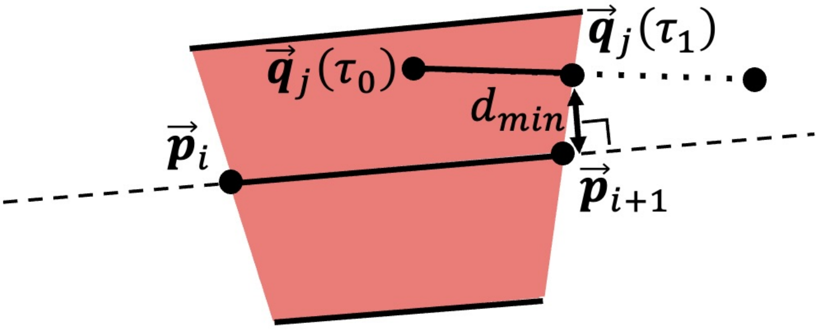

Supplementary Figure 10. Schematic distance minimization procedure described in Algorithm 6, as implemented in Algorithm 5, where $\boldsymbol{a}_0 = \boldsymbol{p}_i$, $\boldsymbol{a}_1 = \boldsymbol{p}_{i+1}$, $\boldsymbol{b}_0 = \boldsymbol{q}_j(\tau_0)$, and $\boldsymbol{b}_1 = \boldsymbol{q}_j(\tau_1)$. Notably, d_{min} falls outside the finite segment $\overline{\boldsymbol{p}_i\boldsymbol{p}_{i+1}}$, necessitating consideration of the infinite line segment $\overleftrightarrow{\boldsymbol{p}_i\boldsymbol{p}_{i+1}}$.

2.4.3 Minimum average direct-flip (MDF) distance

Subsequent swept path selection is guided by minimum average direct-flip (MDF) distance (Algorithm 7 and Equation 3). Given two streamlines P_1 and P_2, each containing N points such that $P_1 = \{\boldsymbol{p}_{1,i}\}_1^N$ and $P_2 = \{\boldsymbol{p}_{2,i}\}_1^N$, MDF distance is the minimum average pairwise euclidean distance across both pairings between the two streamlines [36]:

$$\begin{aligned} d_{direct}(P_1, P_2) = d(P_1, P_2) &= \frac{1}{N}\sum_{i=1}^{N} ||\boldsymbol{p}_{1,i} - \boldsymbol{p}_{2,i}|| \\ d_{flipped}(P_1, P_2) &= d(P_1, P_2^F) = d(P_1^F, P_2) \\ MDF(P_1, P_2) &= \min(d_{direct}(P_1 P_2), d_{flipped}(P_1, P_2)) \end{aligned} \tag{3}$$

where P^F indicates the reversed point sequence defined by a streamline P, such that if $P = \{\boldsymbol{p}_1, \boldsymbol{p}_2, \cdots, \boldsymbol{p}_N\}$, then $P^F = \{\boldsymbol{p}_N, \boldsymbol{p}_{N-1}, \cdots, \boldsymbol{p}_1\}$. Notably, P_1 and P_2 must contain the same number of points to perform the MDF calculation, such that one or both streamlines must be temporarily resampled.

MDF distance is minimized by streamlines that are spatially nearby, parallel, and of similar length. These characteristics ensure that during sweep exclusion (Algorithm 4), subsequent next path selections yield the "nearest" remaining streamline, such that the resulting thinned set is densely packed with an inter-streamline distance larger than but ideally close to the ideal line spacing w_s. Additionally, By starting sweep exclusion with the longest path in the initial streamline set, MDF distance encourages subsequent selections of longer swept streamlines, such that the average toolpath length in the final set is maximized, resulting in a more contiguous print line architecture.

Algorithm 7: MDF-based next path selection

Input : P, the reference streamline
S, a set of N streamlines

Output: the streamline $P_{min} \in S$ with the smallest MDF distance to P

Function `minMDF`(P, S)**:**
  $P_{min} \leftarrow$ random streamline from S
  for each $P_{other} \in S$ **do**
    if MDF(P_{other}, P) $<$ MDF(P_{min}, P) **then**
      $P_{min} \leftarrow P_{other}$
    end
  end
  return P_{min}

2.4.4 Initial path selection

Three initial path selection methods were implemented and compared: shortest-, median-, and longest-first. MDF-guided subsequent path selection favors selection of paths with similar path length. Therefore, it was hypothesized that starting sweep exclusion with the longest path in the set would lead to the highest average path length in the final toolpath set compared to the shortest- and median-first methods. Suppl. Fig. 11 supports this hypothesis, showing that initial path length is correlated with average toolpath length. Furthermore, Suppl. Fig. 11 shows that initial path length is correlated with final toolpath set volumetric coverage. Therefore, longest-first path selection was adopted for the NAATIV3 framework.

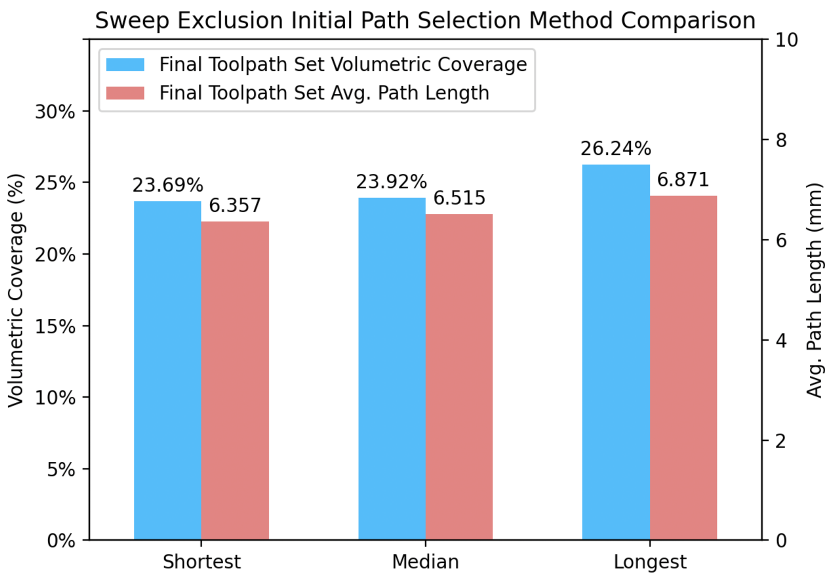

Supplementary Figure 11. Performance comparison between NAATIV3 run with shortest-, median-, and longest-first sweep exclusion processes. Note that any minor discrepancies with Suppl. Fig. 16 are a result of the stochastic nature of the acyclic subgraph selection procedure.

2.5 Cut plane

An optional cut plane step is included in NAATIV3 to reduce the edge density and cycle existence in the constructed dependency graph D, at the cost of potentially reduced material adhesion at the plane location. The necessity and/or specific geometry of the cut plane is based on user judgment. For example, in the cardiac left ventricle case, the helical arrangement of fibers across the ventricle wall results in a large number of cycles in D, which are effectively removed via a semi-vertical plane through the septum (Suppl. Fig. 12). During the cut plane step, a bijective map $C : S_{left} \mapsto S_{right}$ is constructed to track the resulting split components such that all split components $P \in S_{left}$ lie on one side of the cut plane, and all components $P \in S_{right}$ lie on the other side of the cut plane. Delineation between the "left" and "right" side of the cut plane is arbitrary, depending on the specific implementation of the cut plane. During dependency graph population (Algorithm 9), directed edges between streamlines in the domain and range of C are prohibited. In other words, edges of the form $\langle P_i, P_j \rangle$ where $P_i \in S_{left}$ and $P_j \in S_{right}$, or $P_j \in S_{left}$ and $P_i \in S_{right}$, are explicitly prohibited. An overview of the cut plane procedure is provided in Algorithm 8, and the cut plane process for a cardiac ventricle model is shown in Suppl. Fig. 13.

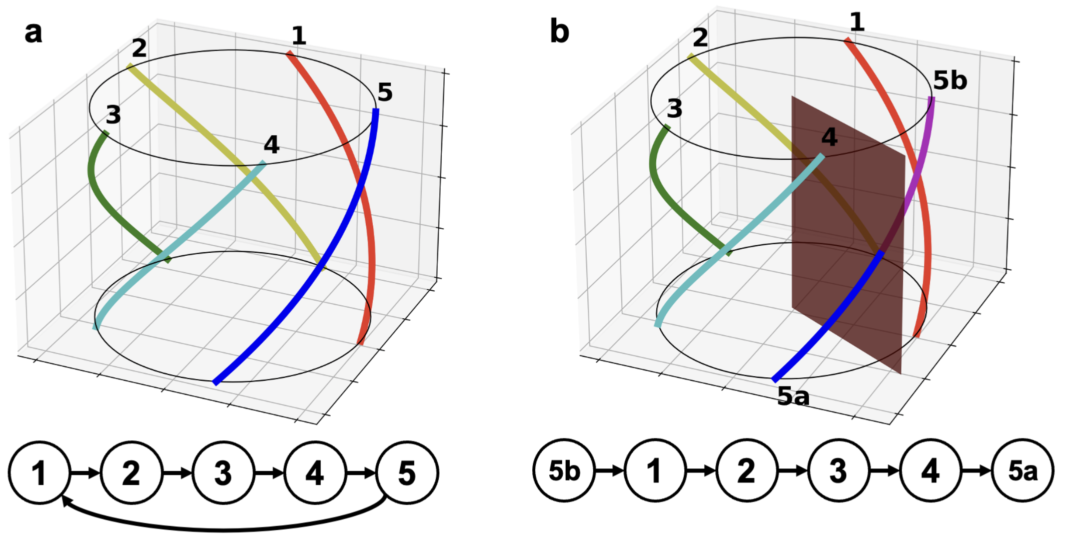

Supplementary Figure 12. Schematic cut plane operation for helically-arranged toolpaths. a) the initial toolpath set before cutting, with associated dependency graph. b) the toolpath set after implementing a vertical cut plane, with dependency between the split components 5a and 5b explicitly prohibited in the dependency graph.

Algorithm 8: Splitting streamlines via a cut plane

Input : $S_{thinned}$, reduced streamline set
`cut_plane`, cut plane geometry

Output: S_{cut}, the cut streamline set
C, the bijective mapping of cut streamlines across the cut plane

Function `planeCut`($S_{thinned}$, `cut_plane`):
    initialize C
    for each $P_i \in S_{thinned}$ **do**
        if P_i *intersects* `cut_plane` **then**
            $P_{i,1}$, $P_{i,2} \leftarrow$ `split`(P_i, `cut_plane`)
            if $P_{i,1}$ *lies on "left" of* `cut_plane` **then**
                $C[P_{i,1}] = P_{i,2}$
            else
                $C[P_{i,2}] = P_{i,1}$
            end
            $S \leftarrow S - \{P_i\}$
            $S \leftarrow S \cup \{P_{i,1}, P_{i,2}\}$
        end
    end
    return S_{cut}, C

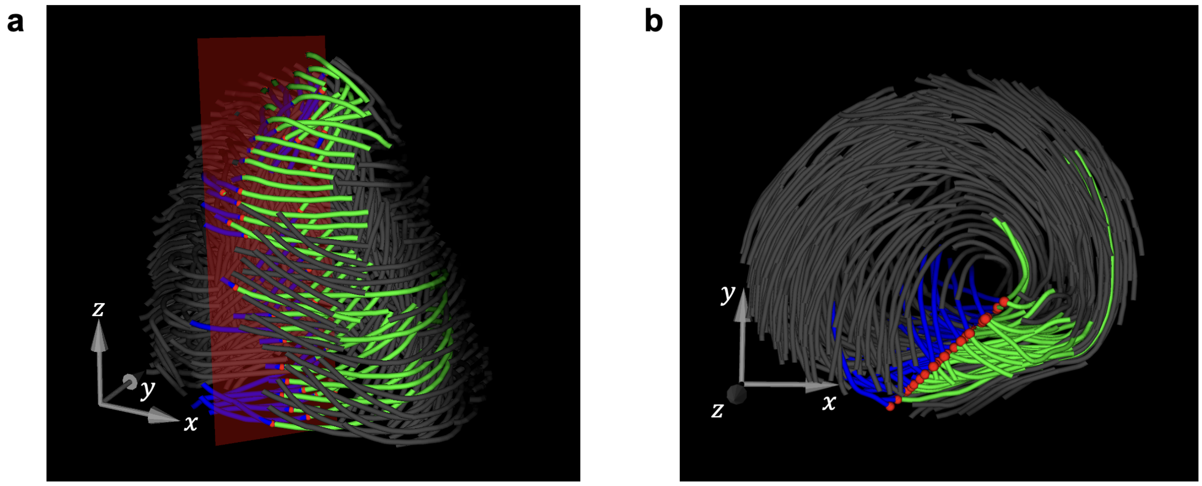

Supplementary Figure 13. Cut plane procedure on a cardiac left ventricle toolpath set. Cut plane and cut points shown in red. Split components on one side of the cut plane shown in blue, with components on the other side shown in green. a) 3D view of the toolpaths. b) top down (+z) view of the toolpaths

2.6 Dependency graph population

NAATIV3 populates the dependency graph D by predicting material/nozzle interference between each directed pair of toolpaths (Algorithm 9). Every edge in D of the form $\langle P_i, P_j \rangle$ denotes that the path P_j must be printed before P_i [27]. In other words, P_j must come before P_i in the final toolpath sequence T. In this way, D must be acyclic for an orderable sequence T to exist (see section 2.7). A volume intersection method is used based on the geometry of the 3D printer's extrusion head and the geometry of deposited material during printing (Algorithm 10). Essentially, if the extruder head would interfere with the previously deposited material along P_i during material extrusion along P_j, then the edge $\langle P_i, P_j \rangle$ is added to the the directed graph D. In this work, a vertical (parallel to the build direction) cylinder is used to model the extrusion head, representing a stainless steel needle (commonly used for 3D bioprinting). The material geometry is determined by the cutting edge of the needle, such that the cross section is effectively elliptical. The exact geometry of the extrusion head and deposited material along each toolpath must correspond to the specific geometry of the physical 3D printer setup. The implementation of Algorithm 10 must be tailored accordingly.

Algorithm 9: Dependency graph population

Input : S, streamline set from which to populate D
C, (optional) the bijective mapping $S_{left} \mapsto S_{right}$ of prohibited dependencies
d_p, print line diameter
d_n, nozzle outer diameter

Output: D, the populated dependency graph

Function `populateDependencyGraph`(S, , d_p, d_n):
  $D \leftarrow (S, \emptyset)$ `// create directed graph with vertices` S `and initially no edges`
  for each $P_i \in S$ **do**
    for each $P_j \in S$ **do**
      if $P_i \neq P_j$ **then** `// no self edges`
        if *C provided AND NOT (($P_i \in S_{left}$ AND $P_j \in S_{right}$) OR ($P_i \in S_{right}$ AND $P_j \in S_{left}$))* **then** `// ensure edge not prohibited via` C
          if `interferencePrediction`(P_i, P_j, d_p, d_n) **then**
            $E(D) \leftarrow E(D) \cup \{\langle P_i, P_j \rangle\}$ `// add directed edge to` D
          end
        end
      end
    end
  end
  return D

In this work, the extruder and material geometries are defined by surfaces termed the "nozzle" and the "material" surfaces, denoted n and m, respectively (Suppl. Fig. 14. n is constructed by integrating the bottommost surface of the extruding needle along the segment of interest in P_j. Similarly, m is constructed by integrating the topmost surface of the deposited material, defined by the cutting edge of the nozzle along the segment of interest in P_j. If $n(x, y) < m(x, y)$ for any point (x, y) in the shared domain $\mathrm{domain}(n) \cap \mathrm{domain}(m)$, then during traversal of P_j, the nozzle would "cut through" the material that was previously deposited along P_i, and the edge $\langle P_i, P_j \rangle$ is added to D. In practice, the construction and evaluation of these surfaces is conducted numerically.

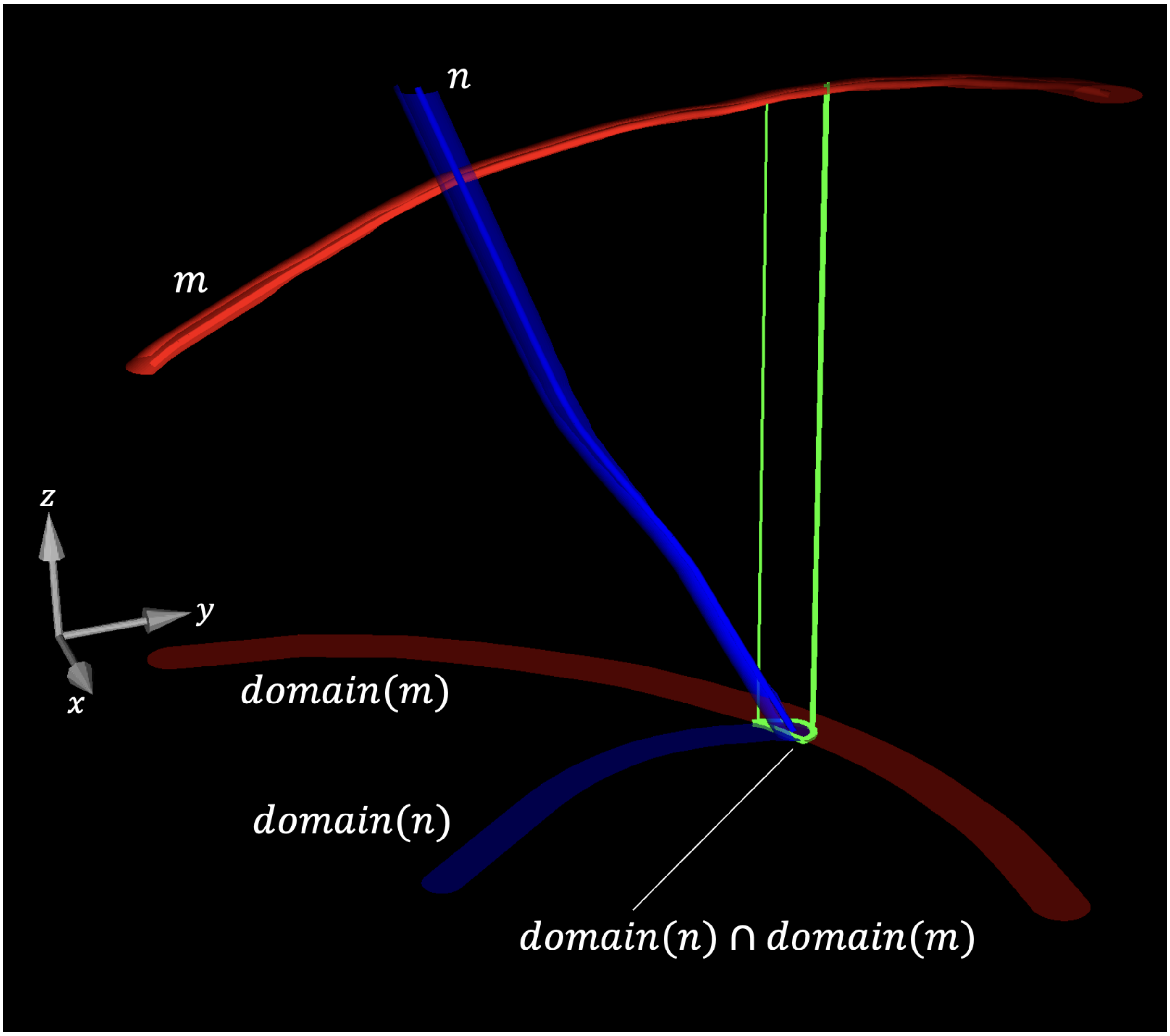

Supplementary Figure 14. Visualization of surface evaluation (Algorithm 10) for two streamlines, with P_1 in red and P_2 in blue. "Material surface" m and $\mathrm{domain}(m)$ for P_1 shown in red, "nozzle surface" n and $\mathrm{domain}(n)$ for P_2 shown in blue. Domain intersection region shown in green, with projections to m. n falls below m in the domain intersection region, so during printing along P_1 (blue), the extruding needle would "cut through" the deposited material along P_2 (red).

Algorithm 10: Interference prediction

Input : P_1, toolpath for which to simulate deposited material
P_2, toolpath for which to simulate nozzle traversal
d_p, print line diameter
d_n, nozzle outer diameter

Output: bool, `true` if depositing material along P_2 after P_1 would result in nozzle/material obstruction, otherwise `false`

Function `interferencePrediction`(P_1, P_2, d_p, d_n):
    $m \leftarrow$ `MaterialSurface`(P_1, d_p)
    $n \leftarrow$ `NozzleSurface`(P_2, d_n)
    if $\text{domain}(m) \cap \text{domain}(n) \neq \emptyset$ **then**
        if $n(x,y) < m(x,y)$ *for any point* $(x,y) \in \text{domain}(m) \cap \text{domain}(n)$ **then**
            return `true`
        end
    end
    return `false`

2.7 Maximum acyclic subgraph selection

A two-step process is used to find a maximum acyclic subgraph of the directed dependency graph D (Algorithm 11). First, a subgraph of D is found such that no length-2 cycles exist (Algorithm 12). Second, an iterative stochastic procedure is used to find the largest acyclic subgraph of the reduced graph (Algorithm 13).

Algorithm 11: Maximum acyclic subgraph selection

Input : D, directed dependency graph
N, iterations for stochastic maximum acylic subgraph selection

Output: $D_{acyclic}$, the maximum found acyclic subgraph of D

Function `maximumAcyclicSubgraph`(D, N):
    `// length-2 cycle (bidirectional edge) removal`
    $D_{reduced} \leftarrow$ `removeBidirectionalEdges`(D)
    `// iterative stochastic acyclic subgraph selection`
    $D_{acyclic} \leftarrow$ `iterativeStochasticAcyclicSubgraph`($D_{reduced}$, N)
    return $D_{acyclic}$

Starting with the directed dependency graph D, vertices (and implicitly any adjacent edges) are removed until no length-2 cycles remain. The vertex with the largest "cycle potential" (CP) is removed during each iteration. Cycle potential is defined by the product of the in-degree and out-degree of a vertex: for a vertex P_i, $CP_i = \deg^+(P_i) \cdot \deg^-(P_i)$. Cycle potential acts as a proxy for "cycle likelihood", denoting the number of unique paths that a cycle could take through a vertex. Vertices with higher cycle potentials are more likely to be involved in a cycle, such that Algorithm 12 aims to find the largest subgraph of D that contains no length-2 cycles.

Algorithm 12: Directed graph bidirectional edge removal

Input : D, directed graph

Output: $D_{reduced}$, the subgraph of D such that no length-2 cycles exist

```
Function removeBidirectionalEdges(D):
    D_reduced ← D
    while any P_i and P_j exist in D_reduced such that ⟨P_i, P_j⟩ ∈ E(D_reduced) and
      ⟨P_j, P_i⟩ ∈ E(D_reduced) do
        P_remove ← null
        CP_max ← 0
        for each vertex P_i ∈ V(D_reduced) do
            for each other vertex P_j ∈ V(D_reduced) − {P_i} do
                if ⟨P_i, P_j⟩ ∈ E(D_reduced) AND ⟨P_j, P_i⟩ ∈ E(D_reduced) then
                    // bidirectional edge
                    CP_i ← deg⁺(P_i) · deg⁻(P_i)
                    CP_j ← deg⁺(P_j) · deg⁻(P_j)
                    if P_remove = null OR CP_i > CP_max OR CP_j > CP_max then
                        if CP_i > CP_j then
                            P_remove ← P_i
                            CP_max ← CP_i
                        else
                            P_remove ← P_j
                            CP_max ← CP_j
                        end
                    end
                end
            end
        end
        V(D_reduced) ← V(D_reduced) − {P_min}
    end
    return D_reduced
```

Algorithm 13 iteratively genenerates random acyclic subgraphs of the reduced graph $D_{reduced}$, returning the largest found subgraph. During each of N iterations, vertices are removed from the graph at random until the graph is acyclic. The largest acyclic subgraph found overall is returned.

Algorithm 13: Iterative stochastic maximum acyclic subgraph selection

Input : D, directed dependency graph
N, iterations for stochastic maximum acylic subgraph selection
Output: $D_{acyclic}$, the maximum found acyclic subgraph of D

Function `iterativeStochasticAcyclicSubgraph`(D, N):
  $D_{acyclic} \leftarrow$ `null`
  `max_size` $\leftarrow 0$
  for $i = 1$ *to* N **do**
    $D_{temp} \leftarrow D_{acyclic}$
    while D_{temp} *is not acyclic* **do**
      $P_{remove} \leftarrow$ random vertex in D_{temp}
      $V(D_{temp}) \leftarrow V(D_{temp}) - \{P_{remove}\}$
    end
    if $|V(D_{temp})| >$ `max_size` **then**
      `max_size` $\leftarrow |V(D_{temp})|$
      $D_{acyclic} \leftarrow D_{temp}$
    end
  end
  return $D_{acyclic}$

2.7.1 Alternative heuristic methods and performance comparison

In addition to the iterative stochastic method described in section 2.7, a heuristic-based method was also developed in an effort to reduce computational costs. This method, deemed Iterative Maximum Cycle Potential removal (IMCP) operates by iteratively removing a vertex from a cyclic graph solely on the CP metric criterion until the graph is acyclic. Specifically, at each iteration, the vertex with the highest CP metric is removed. Similar to the iterative stochastic method, IMCP can also incorporate a length 2-cycle 'preremoval' step. As shown in Suppl. Fig. 15, however, the performance of the IMCP was significantly inferior to that of the iterative stochastic method, such that the iterative stochastic method was ultimately adopted for the NAATIV3 framework. Note also that Suppl. Fig. 15 compares the performance of both the IMCP and iterative stochastic method with and without the length-2 cycle 'preremoval' step, showing that this step improved the performance of both techniques.

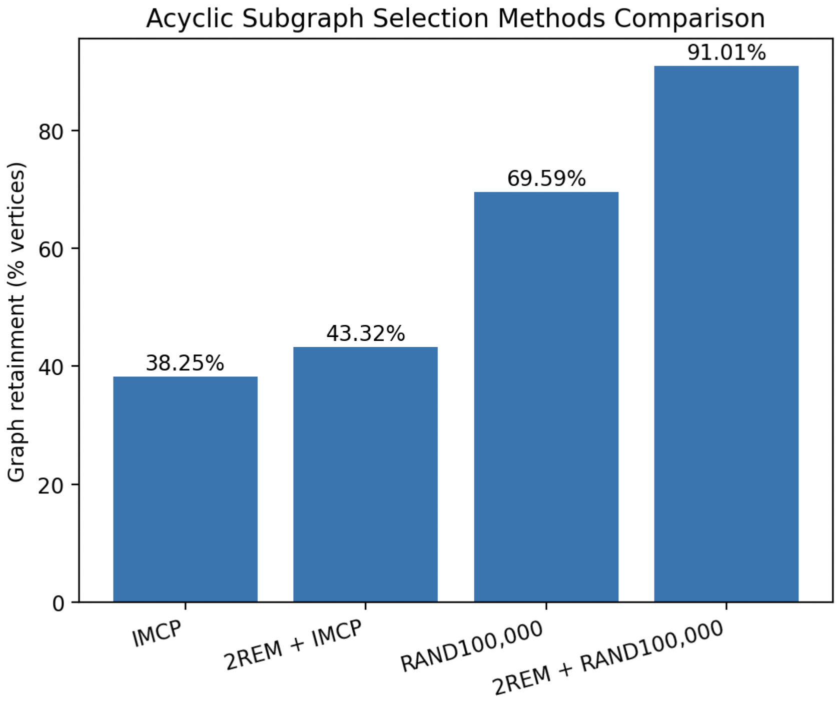

Supplementary Figure 15. Performance comparison of various acyclic subgraph selection techniques. Both the Iterative Maximum Cycle Potential (IMCP) and iterative stochastic (with 100,000 iterations) techniques were tested on a quarter-scale human left ventricle model. Both methods were tested with and without the length-2 cycle preremoval step (2REM).

2.8 Ordering

NAATIV3 employs an action space-restricted greedy search algorithm (Algorithm 14) to order the toolpath set $V(D_{acyclic})$ (the acyclic vertex set of $D_{acyclic}$) into a sequence $T = \{P_i\}_1^{|V(D_{acyclic})|}$ such that total print time is minimized and the resulting sequence T is "printable". Here, printable refers to no expected interference during printing according to the predictive dependency graph $D_{acyclic}$. A toolpath sequence T is printable if for every toolpath $P_i \in T$, P_i does not depend on any subsequent toolpaths in T, i.e. for each $P_i \in T$, there must exist no toolpath $P_j \in T$ for $j \in [i+1, |T|]$ such that $\langle P_i, P_j \rangle \in D_{acyclic}$.

Before executing the search, the point sequence for every toolpath P in $V(D_{acyclic})$ for which $P[-1]_z < P[1]z$ is reversed, such that every toolpath is printed upwards. This operation is desirable for 3D bioprinting and is not strictly necessary. In lieu of this operation, Algorithm 14 can be modified accordingly to consider all paths for traversal in both the forward and backward direction.

The state of each node n in the search tree is defined by an unordered toolpath set $n.U$, and an ordered toolpath sequence $n.T$. The action space for child node traversal is the entire set $n.U$, with each action consisting of removing a path from $n.U$ and appending it to $n.T$. The root node of the search tree n_{root} has an initial unordered set $n_{root}.U$ equal to the entire set $V(D_{acyclic})$ and an initially empty ordered sequence T. As such, with the root node at depth 0 in the search tree, each node at depth d has $|n.U| = |V(D_{acyclic})| - d$ and $|n.T| = d$. It then follows that every node at depth $|V(D_{acyclic})|$ is a solution node, and every solution node must exist at depth $|V(D_{acyclic})|$.

At each expansion of a node, the action set is restricted to those ordered toolpath sequences that retain printability. Consider a node n. The action space for this node is every toolpath $P \in n.U$ for which there does not exist a toolpath $P_{dep} \in n.U - \{P\}$ such that $\langle P, P_{dep} \rangle \in E(D_{acyclic})$. In other words, only paths that depend on no other unprinted paths are considered. This ensures

that in the final toolpath sequence $n_{sol}.T$, there exist no toolpaths pairs P_i, P_j for which $\langle P_i, P_j \rangle \in E(D_{acyclic})$ and $j > i$. Because $D_{acyclic}$ contains no cycles, such a sequence is guaranteed to exist. Algorithm 14 is complete, and therefore a solution will necessarily be found.

The heuristic function used for the greedy search is:

$$H(n) = (|D_{acyclic}| - |n.T|)L_{max} + J(n.T) \tag{4}$$

Where $J(n.T)$ is the total inter-toolpath euclidean travel distance of the sequence $n.T$ given by:

$$J(n.T) = \sum_{i=1}^{|n.T|} ||P_{i-1}[-1] - P_i[1]|| \tag{5}$$

where $P_0[-1]$ for the root node is specified to be at the origin $\langle 0,0,0 \rangle$ and $J(n_{root}.T)$ is defined to be 0. The effect of $H(n)$ is to minimize inter-toolpath travel distance (and therefore travel time) while also guaranteeing that for any node n, its heuristic cost $H(n)$ will necessarily be greater than the heuristic cost of any of its children nodes $H(n_{child})$, and therefore its children will have imminent traversal priority. The child node will possess $|n_{child}.T| = |n.T| + 1$ and $|n_{child}.U| = |n.U| - 1$. It then follows that:

$$\begin{aligned} H(n) - H(n_{child}) &= (|D_{acyclic}| - |n.T|)L_{max} - (|D_{acyclic}| - |n_{child}.T|)L_{max} + J(n.T) - J(n_{child}.T) \\ &= L_{max} + J(n.T) - J(n_{child}.T) \\ &= L_{max} - ||P_{|n.T|}[-1] - P_{|n_{child}.T|}[1]|| \end{aligned} \tag{6}$$

Here, $J(n.T) - J(n_{child}.T)$ denotes the increase of the travel cost function upon traversal to n_{child}. This reduces to the negative euclidean distance between the last point of the last path in $n.T$, $P_{|n.T|}[-1]$, and the first point of the path $P_{|n_{child}.T|}[1]$, which is the path that was appended to $n.T$ to yield $n_{child}.T$ upon traversal. Therefore, if $L_{max} > ||P_{|n.T|}[-1] - P_{|n_{child}.T|}[1]||$, it must be true that $H(n) > H(child)$. To ensure $H(n) > H(n_{child})$, L_{max} must be larger than any possible travel distance between any pair of toolpaths in the set $V(D_{acyclic})$. This achieved by setting L_{max} to the diagonal of the axis-aligned bounding box of the toolpath set $V(D_{acyclic})$. Therefore, for any node n in the search tree, it is guaranteed that for each of its children n_{child}, $H(n_{child}) < H(n)$, and therefore one of its children will have imminent traversal priority.

Next, consider two sibling nodes n_1 and n_2. $n_1.T$ and $n_2.T$ have the same number of paths, and because they share a parent node, the first $|n_1.T| - 1 = |n_2.T| - 1$ paths of $n_1.T$ and $n_2.T$ are identical. The only difference between them is the last path in each sequence (the path that was appended upon expansion of the shared parent node). It then follows that:

$$\begin{aligned} H(n_1) - H(n_2) &= (|D_{acyclic}| - |n_1.T|)L_{max} - (|D_{acyclic}| - |n_2.T|)L_{max} + J(n_1.T) - J(n_2.T) \\ &= J(n_1.T) - J(n_2.T) \end{aligned} \tag{7}$$

such that whichever node has a lower overall travel cost evaluation will also have a lower heuristic cost. Equation 6 and Equation 7 therefore demonstrate that upon traversal of node n, one of its children (if any exist) will necessarily be traversed next. And within the set of children nodes, that with the smallest overall travel cost evaluation will be traversed next. In this way, Algorithm 14 effectively operates as a depth-first search where total travel distance is minimized, while the action space is restricted upon each expansion to ensure the final toolpath sequence $n_{sol}.T$ contains no interference according to the predictive directed graph $D_{acyclic}$.

Algorithm 14: Greedy search toolpath ordering algorithm

Input : $D_{acyclic}$, acyclic directed dependency graph
Output: T, the ordered toolpath sequence

```
Function greedySearch(D_acyclic):
    queue ← PriorityQueue() // ordered according to minimum H
    n_root ← node with n_root.U = V(D_acyclic) and n_root.T = ∅
    queue.add(n_root)
    while queue not empty do
        n ← queue.pop()
        if |n.T| = |U| then // current node is solution
            return n.T
        end
        for each P_i ∈ n.U do
            for each P_j ∈ n.U − {P_i} do
                if ⟨P_i, P_j⟩ ∈ E(D_acyclic) then
                    go to next P_i // do not add to queue
                end
            end
            n_child ← node with n_child.U = n.U − {P_i} and n_child.T = n.T⌢{P_i}
            queue.add(n_child)
        end
    end
```

2.9 Cardiac Toolpathing

In this work, toolpath sets were generated for both a 1:4 (quarter-scale) and a 1:1 (full-scale) cardiac left ventricle model. The full-scale model was obtained from a diffusion MRI scan of a healthy human left ventricle, which was resampled to isotropic 0.8mm voxel size using first-order trilinear interpolation. Importantly, trilinear interpolation is also used to define a continuous field over the discrete diffusion MRI data during tractography (Algorithm 2), such that resampling has no impact on the resulting direction field. Resampling results in higher scan fidelity, which is useful for obtaining a more continuous scan border, yielding higher continuity in tracts near the edge of the region of interest. The resampled scan was manually masked to create the voxelized region of interest M. A diffusion tensor model was fit to the data to extract the primary fiber orientation vector within each voxel, the trilinear interpolation of which provided the input vector field $\boldsymbol{v}(\boldsymbol{x})$. The quarter-scale model was obtained by scaling both M and $\boldsymbol{v}(\boldsymbol{x})$ by a factor of 0.25 along each axis. The input parameters (Table 2) were selected by experimental determination of the print line geometry given the physical printing setup for each model and the manufacturer specifications of the nozzles used. Tractography parameters were set to be proportionate between models.

Supplementary Table 2. NAATIV3 input parameters

Parameter	1:4 scale model	1:1 scale model
n_{seed}	125 $\frac{seeds}{mm^3}$	125 $\frac{seeds}{mm^3}$
l_{max}	18.75 mm	75 mm
Δs	0.125 mm	0.5 mm
w_s	0.548 mm	1.0 mm
d_p	0.548 mm	1.0 mm
d_n	0.4 mm	0.84 mm
N	100,000	100,000,000

2.9.1 Algorithm performance

Volumetric coverage for a toolpath set is defined as the total expected volume of deposited material as a percentage of the total input bounding volume M used to generate that set. For the purposes of this calculation, a circular print line profile along each toolpath is considered, with a diameter equal to the expected print diameter d_p. Volumetric coverage for the thinned streamline set and final toolpath set for both the 1:4 and 1:1 scale cardiac ventricle models is shown in Suppl. Fig. 16.

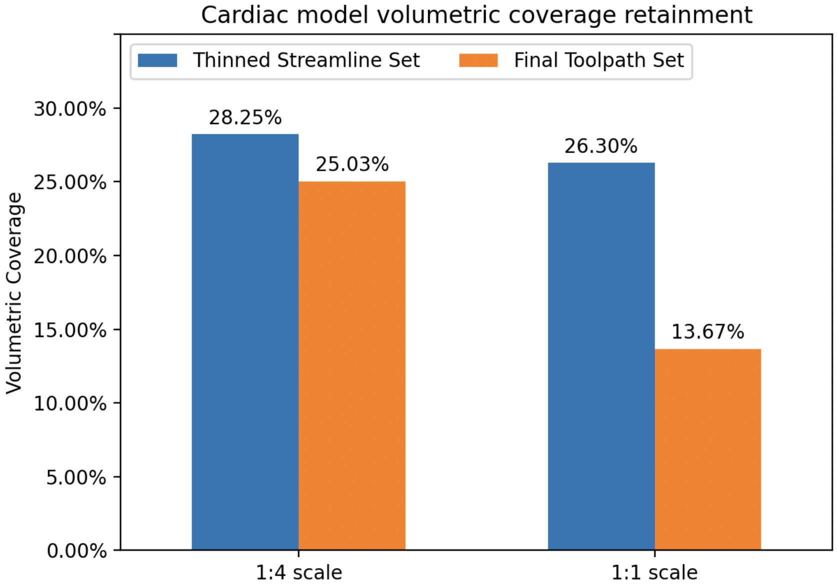

Supplementary Figure 16. Volumetric coverage for the initial thinned streamline set and the final toolpath set for both the 1:4 and 1:1 scale ventricle models.

The 1:4 scale model was processed using a 2023 MacBook Pro with an Apple M2 Max chip featuring 12 CPU cores (8 performance cores @ 3.7 GHz and 4 efficiency cores @ 3.4 GHz) in parallel. The 1:1 scale model was processed using the University of Minnesota Supercomputing Institute, on a Rocky Linux 8.10 partition using 128 CPU cores (AMD EPYC 7763, 2.45 GHz) in parallel. The "response time" (total time elapsed from the start of execution to the end of execution) for each significant step of the algorithm for both the quarter- and full-scale ventricle models are given in Table 3.

Supplementary Table 3. Algorithm per-step run time

Algorithm Step	1:4 scale model	1:1 scale model
Tractography	5m	1h 6m
Sweep exclusion	46m	6d 17h 47m
Dependency graph population	1h 9m	4h 46m
Acyclic subgraph selection	7m[*]	1d 6h 19m[†]

[*]Stochastic iterations: 100,000
[†]Stochastic iterations: 100,000,000

References

[1] B. Carnahan, H. Luther, and J. Wilkes, *Applied Numerical Methods.* Wiley, 1969, ISBN: 9780471135074. [Online]. Available: `https://books.google.com/books?id=IvRQAAAAMAAJ`.
