# Supplementary material for: 3D vector field-guided toolpathing for 3D bioprinting
Source: Commun Eng. 2025 Aug 14;4:154. doi: 10.1038/s44172-025-00489-0 (PMC12354870; doi:10.1038/s44172-025-00489-0)
Supplement: Supplementary file 3 — Description of Additional Supplementary Files [file 44172_2025_489_MOESM3_ESM.pdf]

Description of Additional Supplementary Files

File name: Supplementary Video 1

Description: The complete sweep exclusion process. The original streamline is set shown in blue. The streamlines removed by sweep exclusion are shown in red. The streamlines in the thinned set are shown in green.

File name: Supplementary Video 2

Description: The complete printing process for the 1:4 scale silicone ventricle model. (20× speed)

File name: Supplementary Video 3

Description: A 360° view of the 1:4 scale silicone ventricle model in the clear FRESH support material.

File name: Supplementary Video 4

Description: A 360° view of the 1:1 scale silicone ventricle model in the clear FRESH support material.
